# A Survey of Organ Equivalent and Effective Doses from Diagnostic Radiology Procedures

**DOI:** 10.5402/2013/204346

**Published:** 2012-09-06

**Authors:** Ernest K. Osei, Johnson Darko

**Affiliations:** ^1^Department of Medical Physics, Grand River Regional Cancer Center, Kitchener, ON, Canada N2G 1G3; ^2^Department of Physics and Astronomy, University of Waterloo, Waterloo, ON, Canada N2L 3G1; ^3^Cancer Center of Southeastern Ontario, Kingston General Hospital, Kingston, ON, Canada K7L 5P9; ^4^Department of Oncology, Queens University, Kingston, ON, Canada K7L 5P9

## Abstract

The quantification of radiation risks associated with radiological examinations has been a subject of interest with the increased use of X-rays. Effective dose, which is a risk-weighted measure of radiation to organs in the body associated with radiological examination, is considered a good indicator of radiological risk. We have therefore investigated patient effective doses from radiological examinations. Organ and effective doses were estimated for 94 patients who underwent computed tomography examinations and for 338 patients who had conventional radiography examinations. The OrgDose (version 2) program was used for the estimation of effective doses. The tube potential ranges: 57 kVp to 138 kVp depending on the examination and patient size. The entrance surface doses have a wide range even for the same examination: 0.44–10.31 mGy (abdomen) and 0.66–16.08 mGy (lumbar spine) and the corresponding effective dose ranges 0.025–0.77 mSv and 0.025–0.95 mSv respectively. Effective dose for adult abdomen-pelvic CT examinations ranges 5.4–19.8 mSv with a mean of 13.6 mSv and for pediatrics ranges 2.1–5.5 mSv with a mean of 2.7 mSv. The mean effective dose for adult chest and head CT examinations are 7.9 and 1.8 mSv respectively and for pediatrics are 1.7 and 1.1 mSv.

## 1. Introduction

Diagnostic radiology imaging techniques including conventional radiography, fluoroscopy, and computed tomography (CT) procedures will continue to provide tremendous benefits to modern healthcare and the benefit derived by the patient will far outweigh the small risk associated with any properly conducted imaging modality. Nonetheless, it is important to be able to quantify the risks associated with radiological examinations of patients [1–11]. Access to such information will allow physicians and their patients to better weigh the risks of radiation exposure against the benefits afforded by various radiological examinations and hence make the best informed decisions in terms of options for other diagnostic modalities.

The increase in patients undergoing radiological examinations (especially in CT) has created a great deal of interest in quantifying the risk associated with radiological examinations. Effective dose which is a risk-weighted measure of radiation to organs in the body associated with an examination(s) is considered a good indicator of radiological risk [2–6]. However, it should be realized that effective dose represents a generic estimate of risk from a given procedure for a generic model of the human body [11]. Estimated effective dose from a particular examination can be compared to the naturally occurring background radiation or an alternative imaging examination that provides similar diagnostic information [11]. Use of effective dose will enable comparisons between different types of radiological exposures since it simplifies the complex distribution of dose to various tissues and organs from a particular exposure into a single-dose parameter [3–5, 11]. 

While methods to calculate effective dose have been established [3–5], they depend heavily on the ability to estimate the dose to radiosensitive organs from the radiological procedure(s). The determination of the radiation dose to these organs is very difficult, and direct measurement is not possible. Therefore organ doses are estimated from measurable quantities such as the dose-area product (DAP) or entrance surface dose (ESD) associated with the radiological examination and normalised organ dose data. The Health Protection Agency (HPA) has provided normalised organ doses derived from Monte Carlo modelling of conditions of exposure relevant to 68 common radiographic and fluoroscopic projections [7]. For each projection, normalised doses are presented for 27 organs or tissues. HPA has also provided normalised organ dose data of 23 series of Monte Carlo modelling of conditions of exposure relevant to 27 common models of CT scanners [8–10]. The normalised doses are expressed as absorbed dose in the organ relative to the dose on the axis of rotation of the scanner in the absence of the phantom and expressed in terms of absorbed dose to the International Commission on Radiation Units (ICRU) muscle [12]. The phantom used to generate both Monte Carlo data sets is based upon a mathematical representation of an “average” adult.

Estimated effective doses are generally different even for the same radiological examinations which may be due to the different radiological procedures used at different institutions and hence comparison of dose is very difficult. Patient size, examination technique, and clinical procedures, as well as the skills of the radiographer or radiologist, also affect parameters used in effective dose estimations. Therefore, there has recently been some emphasis on conducting more localized studies of patient dose and associated risk estimate from radiological examinations taking into account the specific machines and departmental protocols that could help in establishing reference levels for monitoring dose from such radiological examinations. Although, the work of previous groups [8, 9, 11, 13–17] certainly provides an excellent resource for evaluating doses from radiological examinations, however since examination protocols varies greatly among various institutions, a local study could provide more relevant information. We have therefore undertaken a study to investigate patients' doses from various radiological examinations. This will help to establish some reference and guidance dose values for radiological examinations performed at this hospital, and would allow us to monitor any changes over time that might arise from aging equipment or changing protocols. It will also allow comparison between the different types of radiological examinations as well as provide us a means to compare doses with that of other hospitals and regions.

## 2. Materials and Methods

### 2.1. Organ and Effective Dose Calculation Using OrgDose

Patient exposure parameters were collected from four conventional X-ray rooms and two computed tomography units at our radiology department. All organ and effective doses were estimated using OrgDose (version 2) computer program [18]. OrgDose has been developed for the estimation of organ and effective doses to patients undergoing medical diagnostic X-ray examinations. It calculates doses from conventional radiography, fluoroscopy, and computed tomography procedures. The OrgDose program uses the normalised organ dose data from Monte Carlo modelling of conditions of exposure relevant to 68common radiographic views [7] and conditions of exposure relevant to 27 common models of CT scanners [10] using a mathematically modelled phantom representing an average adult patient. These data will contain some uncertainty common to all normalized organ dose data using a phantom of a standard reference size. If applied to a patient whose size differs from the phantom used in the derivation of the normalised organ factors, an uncertainty will be introduced into the calculated organ and effective doses [19]. A detailed description of the OrgDose program is published elsewhere [18], however, a brief explanation of how the OrgDose program calculates organ and effective dose is given below.

### 2.2. Conventional Radiography Procedures

Estimation of organ doses from radiographic procedures requires the user to supply a measured or calculated free-in-air entrance surface dose (ESD_ rad_), and techniques parameters used for the examination. The effective dose is calculated as the sum of the weighted equivalent dose in all the tissues and organs of the body as specified in the International Commission on Radiological Protection report 103 (ICRP-103) [3] and report 60 (ICRP-60) [4].

### 2.3. Computed Tomography Procedures

Estimation of organ doses from CT procedures requires the user to supply a measured or estimated free-in-air computed tomography dose index (CTDI_100,air _), tube current (mA), tube rotation time(s), and pitch. The effective dose is calculated as the sum of the weighted equivalent dose in all the tissues and organs of the body as specified in the International Commission on Radiological Protection report 103 (ICRP-103) [3] and report 60 (ICRP-60) [4]. The effective doses to children from CT examinations are estimated by scaling the dose to the “average” adult undergoing similar conditions of exposure by an adult-to-paediatric dose factor. The adult-to-paediatric scaling factor as a function of age is taken from Khursheed et al. [20, 21]. They simulated Monte Carlo calculations of CT examinations on five paediatric phantoms representing children aged 0 (newborn), 1, 5, 10, and 15 years old and an adult phantom representing an “average” adult. This enabled the calculation of relative effective doses to patients of different ages from CT examinations for different parts of the body.

### 2.4. Measurement of Free-in-Air X-ray Machine Output

The outputs of the four conventional X-ray machines used for this study were measured at 100 cm FSD and 80 kVp using calibrated equipment as part of a quality assurance test on the X-ray equipment. The 0.6 cc Farmer chamber (model: Capintec PR06C, Capintec Inc, Ramsey, NJ, USA) was placed at 100 cm from the X-ray source and the collimators were set to approximately 10 × 10 cm^2^ in the plane of the chamber. It was ensured that there was no scattering material close to the setup. The calibration of the farmer chamber together with a Capintec electrometer (Model: Capintec 192, Capintec Inc, Ramsey, NJ, USA) is traceable to an accredited National dosimetry laboratory (NRC, Ottawa, Canada). The outputs of the X-ray machines (mGy/mAs) were determined following the AAPM Task Group no. 61 Protocol [22]. The X-ray machine output, and typical examination parameters such as kV, mA, exposure time, and patient gender are used as input data in the OrgDose program for organ and effective dose estimations.

### 2.5. Measurement of Free-in-Air CTDI_100, air_

The free-in-air CTDI (CTDI_100,air _) for the two scanners (GE Lightspeed VCT and GE Lightspeed Pro 16) were measured according to the recommendation by the EUR 16262EN [6]. The CTDI is a measure of the dose from a single-slice irradiation and it is defined as the integral along a line parallel to the axis of rotation *(z)* of the dose profile *D(z)*, divided by the nominal slice thickness [6]. The CTDI was obtained from a measurement of dose in air, *D(z)*, along the *z*-axis using a 100 mm standard CT ionization chamber (Model Capintec PC-4P, Capintec Inc, Ramsey, NJ, USA) and a capintec electrometer (Model Capintec 192, Capintec Inc, Ramsey, NJ, USA). The calibration of the 100 mm CT ionization chamber together with the Capintec 192 electrometer is traceable to an accredited National dosimetry laboratory (NRC, Ottawa, Canada). The specifications of the 100 mm CT chamber are: nominal volume is 3 mL, wall thickness is 0.3 mm, the diameter is 7.0 mm and chamber length is 102 mm. The CTDI_100,air_ normalized to 100 mAs (mGy/100 mAs), CT scanner manufacturer and model, and typical scanning parameters such as kV, mA, exposure time, pitch, gender, and start and end positions of each scan are used as input data in the OrgDose program for organ and effective dose estimations.

## 3. Results 

Tables 1(a) and 1(b) show a summary of patients' characteristics and the technical parameters used for the various types of examination in this study. A summary of the sample size, applied X-ray tube potential and current-time (mAs) product for conventional radiography examinations and the mean mAs and scan length for computed tomography examinations are also provided. Key statistical parameters of the entrance surface dose and effective dose for simple radiographic examinations is shown in Table 2(a). Table 2(b) shows the estimated effective doses from CT examinations for both adults and paediatrics. A comparison of the estimated entrance surface dose with published data is given in Table 3. Tables 4 and 5 compares the estimated mean effective dose for conventional radiography and computed tomography respectively with published data. Our measured free-in-air CTDI_100_ on the GE Lightspeed VCT and GE Lightspeed Pro 16 both at 120 kVp is 26.56 mGy/100 mAs and 25.89 mGy/100 mAs, respectively. The ImPACT [21] calculator gave values of 27.9 and 26.6 mGy/100 mAs for the GE Lightspeed VCT and GE Lightspeed Pro 16, respectively at 120 kVp.

## 4. Discussion

Diagnostic reference dose levels are a part of the quality criteria as laid down in the European Guidelines on Quality Criteria for Diagnostic Radiographic Images [23]. These are also recommended by the ICRP [5] and by the International Atomic Energy Agency (IAEA) [24] as guidance doses. Diagnostic reference dose values provide quantitative guidance in identifying relatively poor and inadequate use of a technique and a need for appropriate corrective actions. It is therefore imperative that each diagnostic radiology department develop local reference dose levels based on departmental imaging protocols in order to monitor patient doses so as to enable corrective measures where needed. Although reference doses published in other countries or radiological departments can certainly provide excellent resource for evaluating doses from radiological examinations, but since the practices and equipments used may not be comparable with that used at the local institution, a local study could provide more relevant information. We have therefore undertaken this study to develop some local reference dose levels at our radiology department based on our departmental protocols and equipments to help monitor patient exposure from various diagnostic-imaging procedures.

### 4.1. Conventional Radiography Examinations

The applied tube potential, which influences the entrance surface dose and effective dose, revealed a wide range of values even for the same examination. The lowest tube potential was 57 kVp for lateral chest X-ray examination and the highest was 138 kVp also for lateral chest X-ray examination. The entrance surface doses obtained for all the examinations have a wide range: 0.44–10.31 mGy for abdomen examinations and 0.66–16.08 mGy for lumbar spine examinations. The wide range of entrance surface dose for the same examination can be attributed to different X-ray units, exposure factors, image receptors, and, most importantly, variations in patient sizes. We have compared our data with similar work done elsewhere [23–37]. The entrance surface doses presented here are generally lower than published data [25–28], however for a few examinations they are slightly higher. In general, our estimated effective doses are lower than published values [25, 28–30]. Considering the range of entrance surface dose obtained in this study, the mean ESD values for each of the examinations can be used as a basis for a more comprehensive survey. Further investigations could be carried out to establish modified guidance dose levels with our new digital radiography (DR) systems in an effort to further reduce patient dose. As a first step, imaging facilities could aim to achieve entrance surface doses and effective doses below the mean values presented in this paper. In addition, further dose reduction techniques need to be explored in accordance with the principle of ALARA (as low as reasonably achievable) and economic and social factors being taken into account.

### 4.2. Computed Tomography Examinations

We have also compared our mean CT doses with national reference doses in the European Union (EU) [35], UK [36], and Germany [37] and also with other published data in the literature [23, 24, 31–34]. Our estimated mean effective dose from head, chest and abdomen-pelvis CT examinations are lower than the reference doses from EU [35] and Germany [37]. The variation in these doses may be due to differences in imaging protocols and types of equipment. Adult abdomen and pelvic CT examinations appear to have the highest effective dose, ranging from 5.4 mSv to 19.8 mSv with a mean value of 13.6 mSv. Generally, the estimated effective doses from computed tomography examinations are comparable to published results; we estimated a mean effective dose of 1.8 mSv for head CT examinations and Clarke et al. [31]; Tsia [32] and Origgi et al. [33] quoted values of 1.3 mSv, 1.6 mSv, and 1.8 mSv, respectively, for the same examination. The wide range of effective doses for the same examinations in this study could be due to the broad range of mAs and scan lengths employed, which is due to the differences in patient sizes. Estimated effective dose for paediatric CT are well below that of an adult patient for the same examination. We estimated mean effective doses of 7.9 mSv and 13.6 mSv for adult chest and abdomen-pelvis CT examinations, respectively, whereas the corresponding doses for paediatrics CT were 1.7 mSv and 2.7 mSv. Reduction in effective dose from CT examinations can be achieved by reducing the extent of the scan length as much as possible, without missing any vital anatomical regions of interest. Furthermore, reducing the mAs of the examination protocol is also important but this requires a careful consideration of the signal-to-noise in order to avoid significant degradation of image quality and the resulting examination repeats.

The strength of effective dose lies in its utility to estimate and compare the risk from partial body exposures of different anatomic regions and to compare doses from different imaging techniques. According to McCollough et al. [11], the magnitude is nominally equivalent to the dose level that, if applied to the whole body, would result in the same risk as the partial body irradiation being evaluated. However, since effective dose takes into account estimates of relative biologic risk which have evolved over time, and is not a physical parameter that can be directly measured or verified, a true value for the effective dose from an examination does not exist. Thus, any discussion of effective dose must recognize that it is only a broad, generic estimate of risk, and that differences of several mSv do not imply any true differences in biologic risk [11]. Any estimated value reflects the risk of the examination and not the risk to any specific individual, since the weighting coefficients are averaged over age and gender and several assumptions and simplifications are taken into consideration during effective dose determination [11].

## 5. Conclusion

It is very important in the diagnostic radiology departments to monitor and control doses to patients during imaging procedures. The doses delivered to patients in any medical imaging procedure should always be optimized for the given purpose. Representative measurements of the entrance surface dose and effective dose from various examinations should periodically be undertaken, as an essential part of the medical audit and quality assurance programme in any radiology department. Results from our study suggest that there may be room for further dose reduction during X-ray examinations. 

## Figures and Tables

**Table:** (a)

		Patient age		Number of patients			AP thickness		Examination technique parameters			
Projection	Examination	(years)					(cm)		kVp		mAs	
		Range	Mean	Total	Male	Female	Range	Mean	Range	Mean	Range	Mean

AP	Abdomen	25–89	60.5	31	19	12	11–43	23.3	65–90	87.6	10–121	34.4
AP	Cervical spine	16–61	37.7	3	2	1	7–16	11.7	70–80	73.3	4.4–37.7	20.4
Lateral	Cervical spine	16–61	37.0	5	4	1	8–16	13.9	70–80	74.0	7.3–39.6	24.5
** **	Cervical spine*	**16–61**	**37.3**	**8**	**6**	**2**	**7–16**	**13.1**	**70–80**	**73.8**	**4.4–39.6**	**23.0**
Lateral	Chest	15–88	59.8	85	46	39	14–44	32.4	57–138	119.6	3.2–97	34.1
PA	Chest	15–88	60.1	76	38	38	11–38	24.6	70–129	119.1	1.14–18	5.4
** **	Chest*	**15–88**	**60.0**	**161**	**84**	**77**	**11–44**	**28.7**	**57–138**	**119.4**	**1.14–97**	**20.5**
Lateral	Head	40–80	57.6	5	5	0	15.5–17	16.2	70–80	72.8	11.7–53	23.3
PA	Head	40–80	54.2	5	4	1	16–24	20.6	76–120	86.4	10.4–64.7	40.2
** **	Head*	**40–80**	**55.9**	**10**	**9**	**1**	**15.5–24**	**18.4**	**70–120**	**79.6**	**10.4–64.7**	**31.8**
AP	Hip	45–85	63.9	30	10	20	11–34	19.7	73–96	84.6	6–64	19.2
AP	Lumbar spine	14–84	56.6	22	6	16	18–33	27.0	80–110	87.4	12.3–187	90.1
Lateral	Lumbar spine	14–84	59.2	29	8	21	24–43	33.6	90–110	97.3	27.7–243	108.5
Oblique	Lumbar spine	14–76	54.2	12	4	8	18–31	24.7	80–90	82.2	13.6–83.4	54.5
** **	Lumbar spine*	**14–84**	**57.3**	**63**	**18**	**45**	**18–43**	**29.6**	**80–110**	**91.0**	**12.3–243**	**91.8**
AP	Pelvis	14–86	64.5	24	12	12	14–32	21.1	80–103	85.9	6.3–97.2	30.9
AP	Shoulder	80–84	82.2	6	6	0	16–21	17.7	76–76	76.0	3.6–19.9	7.8
AP	Thoracic spine	58–68	62.3	3	1	2	21–32	27.7	70–75	71.7	27–89	58.3
Lateral	Thoracic spine	58–68	63.0	2	0	2	30–32	31.0	75–75	75.0	36–36	36.0
** **	Thoracic spine*	**58–68**	**62.6**	**5**	**1**	**4**	**21–32**	**29.0**	**70–75**	**73.0**	**27–89**	**49.4**

Total		14–89	60.0	338	165	173	—	—	—	—	—	—

^∗^Values are the results of all the various projections of the examination.

**Table:** (b)

	Examination	Patient age (years)		No of patients		Mean mAs		Scan length (cm)	
		Range	Mean	Male	Female	Range	Mean	Range	Mean
	Abdomen and pelvis	21–87	51.3	9	11	70–333.4	187.3	37.4–51.6	46.7
Adults	Chest	38–89	67.2	10	10	64.9–312.6	156.1	25.7–34.3	30.8
	Head	**15–92**	**60.3**	**9**	**11**	194.0–359.1	305.2	13.5–16.1	14.3

	Total*	21–92	59.6	28	32	—	—	—	—

	Abdomen and pelvis	2–9	5.2	10	2	20.7–52.1	26.6	24.8–37.0	28.8
Pediatrics	Chest	1–8	3.8	4	7	20.0–33.6	22.9	12.6–21.8	16.2
	Head	1–9	3.9	9	2	84.0–192.0	128.4	9.6–14.8	12.8

	Total*	**1–9**	**4.3**	**23**	**11**	—	—	—	—

^∗^Values are the results of all the various projections of the examination.

**Table:** (a)

Projection	Examination	Entrance Surface Dose (mGy)		Effective dose (mSv)^$^	
		Range	Mean	Range	Mean
AP	Abdomen	0.44–10.31	1.82	0.025–0.77	0.14
AP	Cervical spine	0.12–1.01	0.62	0.0046–0.037	0.023
Lateral	Cervical spine	0.20–0.61	0.44	0.0019–0.0034	0.0025
	Cervical spine*	**0.12**–**1.01**	**0.50**	**0.0019**–**0.037**	**0.0103**
Lateral	Chest	0.02–3.02	0.94	0.0012–0.33	0.11
PA	Chest	0.03–0.48	0.14	0.0026–0.071	0.0204
	Chest*	**0.02**–**3.02**	**0.57**	0.0012–0.33	0.066
Lateral	Head	0.32–2.02	0.76	0.0027–0.0204	0.0071
PA	Head	0.36–2.93	1.67	0.0037–0.048	0.0202
	Head*	**0.32**–**2.93**	**1.22**	0.0027–0.048	0.014
AP	Hip	0.19–3.60	0.87	0.0078–0.16	0.034
AP	Lumbar spine	0.66–10.02	3.72	0.055–0.95	0.38
Lateral	Lumbar spine	1.28–16.08	6.28	0.025–0.39	0.13
Oblique	Lumbar spine	0.73–3.22	2.41	0.06–0.13	0.093
	Lumbar spine*	**0.66**–**16.08**	**4.65**	0.025–0.95	0.21
AP	Pelvis	0.36–5.64	1.57	0.040–0.65	0.16
AP	Shoulder	0.11–0.63	0.25	0.00082–0.0047	0.0019
AP	Thoracic spine	1.08–3.56	2.21	0.11–0.35	0.22
Lateral	Thoracic spine	1.65	1.65	0.032–0.31	0.32
	Thoracic spine*	**1.08**–**3.56**	**1.99**	**0.032**–**0.35**	**0.14**

^∗^Values are the results of all the various projections of the examination.

^$^All effective dose data are rounded to 2 significant figures.

**Table:** (b)

	Examination	Effective dose (mSv)	
		Range	Mean
Adults	Abdomen and pelvis	5.4–19.8	13.6
	Chest	3.6–13.8	7.9
	Head	1.1–2.5	1.8

Pediatrics	Abdomen and pelvis	2.1–5.5	2.7
	Chest	1.2–2.8	1.7
	Head	0.9–1.3	1.1

**Table 3:** Estimated entrance surface dose (ESD) for all projections and examinations compared with reported values in the literature.

Projection	Examination	Entrance surface dose (mGy)					
		This work	Reference [28]	Reference [27]	Reference [25]^$^	Reference [26]	
			Mean	Mean	Mean	Range	Mean
AP	Abdomen	1.82	2.47	6		0.21–7.21	2.67
AP	Cervical spine	0.62	—	—	1.30	—	—
Lateral	Cervical spine	0.44	—	—	1.03	—	—
	Cervical spine*	**0.50**	—	—	—	—	—
Lateral	Chest	0.94	0.20	1.0	0.30	0.12–1.48	0.56
PA	Chest	0.14	0.11	0.2	0.33	0.02–0.38	0.17
	Chest*	**0.57**	—	—	—	—	—
Lateral	Head	0.76	1.13	1.5	0.95	0.54–2.08	1.13
AP/PA	Head	1.67	1.64	3	1.15	0.62–2.68	1.57
	Head*	**1.22**	—	—	—	—	—
AP	Hip	0.87	—	—	—	—	—
AP	Lumbar Spine	3.72	2.57	6	2.77	0.96–7.21	3.05
Lateral	Lumbar Spine	6.28	5.41	14	4.43	0.59–17.66	7.84
Oblique	Lumbar Spine	2.41	—	—	—	—	—
	Lumbar spine*	**4.65**	—	—	—	—	—
AP	Pelvis	1.57	1.84	4	2.08	0.91–7.02	2.86
AP	Shoulder	0.25	—	—	—	—	—
AP	Thoracic Spine	2.21	—	3.5	1.53	—	—
Lateral	Thoracic Spine	1.65	—	10.0	—	—	—
	Thoracic spine*	**1.99**	—	—	—	—	—

^∗^Values are the results of all the various projections of the examination.

^$^The average of three hospitals.

**Table 4:** Estimated mean effective dose for all examinations and projections compared with reported values in the literature.

Projection	Examination	Estimated mean effective dose (mSv)				
		This work^$^	Reference [30]	Reference [28]	Reference [29]	Reference [25]^&^
AP	Abdomen	0.14	—	—	0.7	—
AP	Cervical spine	0.023	—	—	—	0.06
Lateral	Cervical spine	0.0025	—	—	—	<0.01
	Cervical spine*	**0.0103**	—	—	—	—
Lateral	Chest	0.11	—	—	0.04	0.03
PA	Chest	0.0204	—	—	0.02	0.04
	Chest*	**0.066**	0.02	0.04	—	—
Lateral	Head	0.0071	—	—	0.01	0.01
AP/PA	Head	0.0202	—	—	0.03	0.01
	Head*	**0.014**	0.04	0.03	—	—
AP	Hip	0.034	—	—	—	—
AP	Lumbar spine	0.38	—	—	0.7	0.28
Lateral	Lumbar spine	0.13	—	—	0.3	0.04
Oblique	Lumbar spine	0.093	—	—	—	—
	Lumbar spine*	**0.21**	1.3	0.48	—	—
AP	Pelvis	0.16	0.7	0.33	0.7	0.29
AP	Shoulder	0.0019	—	—	—	—
AP	Thoracic spine	0.22	—	—	0.4	0.14
Lateral	Thoracic spine	0.32	—	—	0.3	—
	Thoracic spine*	**0.14**	0.7	—	—	—

^∗^Values are the results of all the various projections of the examination.

^$^All data are rounded to 2 significant figures.

^&^The average of three hospitals.

**Table 5:** Estimated effective dose from computed tomography examinations of the adult patient compared with reported values in the literature.

Reference	Examination	mAs		Scanned length (cm)		Effective dose (mSv)	
		Range	Mean	Range	Mean	Range	Mean
This work	Chest	64.9–312.6	156.1	25.7–34.3	30.8	3.6–13.8	7.9
	Head	194.0–359.1	305.2	13.5–16.1	14.3	1.1–2.5	1.8
	Abdomen and pelvis	70–333.4	187.3	37.4–51.6	46.7	5.4–19.8	13.6

Reference [31]	Chest	33–260	153.3	13.4–26.1	20.7	3.8–9.2	5.6
	Head	320–450	370	13.5	13.5	1.0–1.5	1.3
	Pelvis	125–450	305	13.5–17.5	15.5	1.9–8.2	5.8
	Abdomen	150–340	231.3	13.8–24.6	18.5	3.8–9.5	5.8
	Abdomen and pelvis*	—	—	—	—	—	12.3

Reference [32]	Chest	40–1350	268	6–45	22.1	—	8.4
	Head	90–1500	343	7.5–21	12.2	—	1.6
	Pelvis	60–1350	295	7.5–40	18.7	—	7.7
	Abdomen	60–1575	292	10–35	20.4	—	7.4
	Abdomen and pelvis*	—	—	—	—	—	15.1

Reference [33]	Chest	120–350	231.6	10–40	23.8	2.8–16.0	7.9
	Head	220–580	367.3	7.5–20	12.7	0.6 – 4.1	1.8
	Pelvis	150–560	281.7	7.5–40	18.7	2.9–35.0	8.8
	Abdomen	175–650	281.8	7.5–41.4	22.2	2.3–20.0	7.9
	Abdomen and pelvis*	—	—	—	—	—	16.7

Reference [34]	Chest	—	—	—	—	3.8–26.0	9.3
	Head	—	—	—	—	1.7–4.9	2.8
	Pelvis	—	—	—	—	3.5–15.5	9.0
	Abdomen	—	—	—	—	3.6–26.5	10.1
	Abdomen and pelvis	—	—	—	—	7.3–31.5	16.5

Reference [35]	Chest	—	—	—	—	—	8.8
	Head	—	—	—	—	—	2.0
	Pelvis	—	—	—	—	—	6.6
	Abdomen	—	—	—	—	—	9.0
	Abdomen and pelvis*	—	—	—	—	—	15.6

Reference [36]	Chest	—	—	—	—	—	5.8
	Head	—	—	—	—	—	1.5
	Pelvis	—	—	—	—	—	—
	Abdomen	—	—	—	—	—	5.3
	Abdomen and pelvis	—	—	—	—	—	7.1

Reference [37]	Chest	—	—	—	—	—	5.7
	Head	—	—	—	—	—	2.8
	Pelvis	—	—	—	—	—	7.2
	Abdomen	—	—	—	—	—	—
	Abdomen and pelvis	—	—	—	—	—	14.4

^∗^Estimated effective dose is the sum of the abdomen and pelvis data.
